# Effect of 17β-Estradiol, Progesterone, and Tamoxifen on Neurons Infected with *Toxoplasma gondii* In Vitro

**DOI:** 10.3390/microorganisms9102174

**Published:** 2021-10-19

**Authors:** María de la Luz Galván Ramírez, Judith Marcela Dueñas-Jiménez, Adrián Fernando Gutiérrez-Maldonado, Laura Rocío Rodríguez Pérez

**Affiliations:** 1 Departamento de Microbiología y Patología, Centro Universitario de Ciencias de la Salud, Universidad de Guadalajara, Guadalajara 44340, Mexico; rocio2427@hotmail.com; 2 Laboratorio de Neurofisiología, Departamento de Fisiología, Centro Universitario de Ciencias de la Salud, Universidad de Guadalajara, Sierra Mojada 950, Col. Independencia, Guadalajara 44340, Mexico; judithmarceladuenas@gmail.com; 3 Coordinación Carreras Agronegocios y Agrobiotecnología, Centro Universitario de la Ciénega, Universidad de Guadalajara, Av. Universidad, no.1115, col. Lindavista, Ocotlán 47810, Mexico; fernando.gutierrez@cuci.udg.mx

**Keywords:** *Toxoplasma* infection, neurons, 17β-estradiol, progesterone, agonists, antagonists, cerebral toxoplasmosis

## Abstract

*Toxoplasma gondii* (*T. gondii*) is the causal agent of toxoplasmosis, which produces damage in the central nervous system (CNS). *Toxoplasma*–CNS interaction is critical for the development of disease symptoms. *T. gondii* can form cysts in the CNS; however, neurons are more resistant to this infection than astrocytes. The probable mechanism for neuron resistance is a permanent state of neurons in the interface, avoiding the replication of intracellular parasites. Steroids regulate the formation of *Toxoplasma* cysts in mice brains. 17β-estradiol and progesterone also participate in the control of *Toxoplasma* infection in glial cells in vitro. The aim of this study was to evaluate the effects of 17β-estradiol, progesterone, and their specific agonists–antagonists on *Toxoplasma* infection in neurons in vitro. Neurons cultured were pretreated for 48 h with 17β-estradiol or progesterone at 10, 20, 40, 80, or 160 nM/mL or tamoxifen 1 μM/mL plus 17β-estradiol at 10, 20, 40, 80, and 160 nM/mL. In other conditions, the neurons were pretreated during 48 h with 4,4′,4″-(4-propyl-[1H] pyrozole-1,3,5-triyl) trisphenol or 23-bis(4-hydroxyphenyl) propionitrile at 1 nM/mL, and mifepristone 1 µM/mL plus progesterone at 10, 20, 40, 80, and 160 nM/mL. Neurons were infected with 5000 tachyzoites of the *T. gondii* strain RH. The effect of 17β estradiol, progesterone, their agonists, or antagonists on *Toxoplasma* infection in neurons was evaluated at 24 and 48 h by immunocytochemistry. *T. gondii* replication was measured with the 3-(4,5-dimethylthiazol-2-yl)-2,5-diphenyltetrazolium bromide reduction assay. 17β-Estradiol alone or plus tamoxifen reduced infected neurons (50%) compared to the control at 48 h. Progesterone plus estradiol decreased the number of intracellular parasites at 48 h of treatment compared to the control (*p* < 0.001). 4,4′,4″-(4-propyl-[1H] pyrozole-1,3,5-triyl) trisphenol and 23-bis(4-hydroxyphenyl) propionitrile reduced infected neurons at 48 h of treatment significantly compared to the control (*p* < 0.05 and *p* < 0.001, respectively). The *Toxoplasma* infection process was decreased by the effect of 17β-estradiol alone or combined with tamoxifen or progesterone in neurons in vitro. These results suggest the essential participation of progesterone and estradiol and their classical receptors in the regulation of *T. gondii* neuron infection.

## 1. Introduction

*Toxoplasma gondii* is an intracellular obligate protozoan. It is the causal agent of toxoplasmosis, which induces damage in the central nervous system. Its success as an invasive organism is that it possesses a great capacity to migrate transepithelial [1]. When *Toxoplasma gondii* reaches essential organs, such as the brain, it causes cerebral toxoplasmosis. Neurons are the main objective of *Toxoplasma gondii* at the central nervous system (CNS) in vivo, and this parasite–neuron interaction promotes the establishment of chronic CNS infection [2]. *Toxoplasma gondii* can maintain itself during its entire lifespan within the CNS in a bradyzoite stage encysted in neurons [3,4,5]. Furthermore, neurons derived from stem cells can also develop cysts of *Toxoplasma* [6]. However, *Toxoplasma* division in neurons is not an active process compared to glial cells [7]. In congenital toxoplasmosis, neurologic forms can cause hydrocephalus and microcephaly. The classic triad is hydrocephalus, ventricular dilation, and intracerebral calcifications. Brain toxoplasmosis too can course with meningoencephalitis, psychiatric complications, personality changes, decreased IQ, and psychomotor performance, and it is also associated with schizophrenia [8,9].

On the other hand, there are sex differences between female and male BALB/K mouse infected with *Toxoplasma*. Females exhibit higher necrotic lesions, mortality, and parasite burden than males [10,11]. The administration of testosterone to females reduced parasite burdens and lesions [12]. Furthermore, dehydroepiandrosterone (DHEA) can reduce the passive invasion and viability of extracellular *Toxoplasma* tachyzoites in vitro [13]. These results suggest the participation of an essential steroid hormone in *Toxoplasma gondii* infection.

17β-estradiol (E2). 17β-estradiol belongs to a group of hormones synthesized in the CNS derived from cholesterol. The main functions of E2 in the CNS are activating genomic effects typically related with classical estrogen receptors (ERα, ERβ). The non-genomic response of E2 is through membrane receptors (GPERs) that generate fast cellular signaling pathways, inducing the activity of secondary messengers and kinase proteins [14,15,16,17,18,19,20].

Agonists of E2 (PPT and DPN). Among the estrogen receptor (ER) agonists, there is a selective ligand, such as 4,4′,4″-(4-propyl-[1H]) pyrazole-1,3,5-triyl) trisphenol (PPT), which is specific to the alpha estradiol receptor (ERα). There, too, is a selective agonist for the 17-beta estradiol receptor (ERβ): 23-bis(4-hydroxyphenyl) propionitrile (DPN) [21]. Transcription of ERs is stimulated by PPT and DPN, explaining their action mechanisms [22]. DPN possesses a greater affinity for ERβ, with a 100-fold greater preference towards ERβ, being 30–50-fold more potent as an agonist for ERβ than for ERα. PPT exhibits high affinity for ERα, with a 400-fold greater preference for ERα, being 1000-fold more potent as an agonist for ERα than ERβ; thus, it is used to evaluate the biological role of ERα [23].

Tamoxifen (Tam) is the prototype of a class of pharmaceuticals, known as selective estrogen receptor modulators (SERMs), that are known exert estrogenic and anti-estrogenic effects on various tissues. This pharmaceutical drug competes with 17β-estradiol for binding to ER, with an affinity 100–1000-fold greater than that of estradiol. The ER–tamoxifen complex binds to DNA; however, estrogens’ agonist and antagonist messages act through the presentation of promoting elements dependent on the cell type [21,24,25,26].

Progesterone (P4) is synthesized within the CNS, both in neurons and glial cells; thus, it can be considered a neurosteroid [27,28]. The specific receptors of progesterone include progesterone receptor A (PRA) and progesterone receptor B (PRB) [26] as well as several progesterone membrane receptors. The neurons and glial cells synthesize progesterone as a neurosteroid [27,28].

Mifepristone (RU 486) was the first progesterone antagonist. In the presence of progesterone, mifepristone (Mif) acts as a competitive antagonist of PRA. In the absence of progesterone, Mif acts as a partial antagonist of PRB [29,30,31,32]. On the other hand, the progesterone and the antagonist RU 486 can modulate the cells’ autophagy and is strongly associated with cellular survival [33,34,35].

17β-estradiol and progesterone in neurons. In 1986, Pung and Luster demonstrated that in ovariectomized rats, there were variations in susceptibility to infection when hormone levels are re-established [36,37]. Despite the significant damage caused by the *T. gondii* parasite in the CNS, there are few studies on 17β-estradiol’s and progesterone’s (P4) effects on cerebral toxoplasmosis [38].

Recently our research group demonstrated that *T. gondii* induces the expression of ERα and ERβ and lowers prolactin receptor (PRLR) in THP-1 monocytes cells. Progesterone decreases PRLR and ERβ and increases ERα; E2 decreases PRLR, and prolactin (PRL) reduces ERα and ERβ expression [39]. However, the effect of estradiol and progesterone on cultured neurons infected with *Toxoplasma* is unknown. This study aimed to determine the role of 17β-estradiol and progesterone as well as agonists (i.e., DPN and PPT) and antagonists (i.e., tamoxifen and mifepristone) on neurons infected by *Toxoplasma gondii* in vitro.

## 2. Material and Methods

### 2.1. Ethical Aspects

This study was carried out from January 2019 to March 2020 in strict accordance with the recommendations of Official Mexican Standards NOM-067. The protocol was approved with registration number CI-07618. All animal experiments were approved by the biosafety, research, and ethics committees of the University Center of Health Sciences of the University of Guadalajara.

### 2.2. Parasites

We used tachyzoites of the virulent RH strain of *Toxoplasma gondii* for our experiments. They were obtained by intraperitoneal (i.p.) passage in Swiss–Webster female mice of six-week-olds, injected with 100,000 tachyzoites. After three days, parasites were harvested from peritoneal exudates to infect neurons. The concentration of tachyzoites was determined by counting in a hemocytometer using a light microscope [39].

### 2.3. Cortical Neuron Culture

For the culture of neurons, we sacrificed one to three newborn rats not more than four days old. We used the brain cortex. The brain tissue was obtained by craniotomy and mechanically dissociated in OPTI-MEM 51985 medium (Invitrogen, Carlsbad, CA, USA) supplemented with 10% horse serum (HS) and a 1% solution of penicillin–streptomycin. Cells were counted using a 0.3% Trypan Blue solution to determine the percentage of survival. The cells were plated (50,000) onto 0.1% poly-L-lysine pretreated coverslips placed in 24-well plates and maintained at 37 °C in a 95% air and 5% CO_2_ atmosphere. The medium was changed every two days until obtaining 90% confluence.

### 2.4. Neuron Separation

After seven or eight days of culture, 90% of confluent cells were separated (oligodendrocytes, microglia, and astrocytes). Then neurons were incubated in Neurobasal^®^ medium-plus B27 supplement plus cytosine arabinoside 5 µM for 24 h. The medium was replaced every three days. The neurons obtained had 95% purity.

### 2.5. Experimental Treatments in Neurons In Vitro

We add to neurons 10, 20, 40, 80, or 160 nM/mL of E2 or P4 for 48 h or the combination of E2 and P4 at a concentration of 160 nM/mL. Tam was utilized at a concentration of 1 μM/mL plus E2 10, 20, 40, 80, or 160 nM/mL. PPT or DPN was administrated to the cultured neurons at a concentration of 1 nM/mL. Mif was added at a dose of 1 µM/mL plus P4 at doses of 10, 20, 40, 80, and 160 nM/mL; then, *T. gondii* tachyzoites were added (5 × 10^3^ parasites per well), and they were allowed to infect the neurons for periods of 24 and 48 h. Each hormone dose was evaluated independently in three replications, repeating the experiment three times. For the immunocytochemical experiment, infected and uninfected cells were fixed with 3.7% paraformaldehyde, for 5 min at room temperature and stored at 4 °C.

### 2.6. Immunocytochemical Method

We detected *T. gondii* infection in cultured neurons by immunocytochemistry. Neurons were washed with phosphate-buffered solution (0.1 M PBS), and they were permeabilized with Triton X-100 in PBS (TPBS) for 1 h. Next, the neurons were incubated with mouse anti-β-Tubulin (β-Tubulin) antibody (1:450, MAB1637); Millipore, Carpinteria, CA, USA) and rabbit anti-*Toxoplasma* antibody (1:1500; GenWay Biotech, San Diego, CA, USA) diluted and incubated at 4 °C for 16 h in a humidity chamber. Then, cells were incubated with Alexa Fluor 594 antibody-labeled anti-mouse IgG (1:1000, Abcam 150116) and Alexa Flour 488 antibody-labeled anti-rabbit IgG (1:1000, Abcam 150073) for one hour at room temperature under conditions of darkness in a humidity chamber. Finally, the cells were washed twice with PBS and incubated for 5 min with a nucleic acid stain (DAPI, Invitrogen) diluted 1:3000 in PBS.

### 2.7. Microscopic Analysis

The neurons were quantified a total of nine times for each hormone dose and their agonists and antagonists. We counted one hundred cells for each dose: the number of infected neurons was evaluated using digital images from an Olympus IX71 microscope (40× magnification) employing Image-Pro Plus ver.6.0 software (Media Cybernetics 2.6) to merge the images.

### 2.8. T. gondii Replication

The enzyme mitochondrial succinate dehydrogenase develops the MTT Tetrazolium reduction assay in live cells. Only viable and early apoptotic cells can reduce the tetrazolium salt MTT (yellow), resulting in the formation of water-insoluble formazan crystals (purple); dead cells will therefore retain the yellow color of the medium [40,41,42].

*T. gondii* replication in infected and treated neurons was evaluated by the MTT assay. We used 96-well plates to culture neurons (5 × 10^4^ cells/1.7 cm^2^) using the method described previously. Forty microliters of MTT solution (5 mg/mL; Sigma, St. Louis, MO, USA) were added for two h and incubated at 37 °C in 5% CO_2_ and 95% air. Subsequently, we added 100 µL of 50% dimethylformamide and 20% sodium dodecyl sulfate (SDS) to dissolve the formazan crystals, and the solution was incubated overnight. Absorbance was measured at 570 nm in an enzyme-linked immunosorbent assay (ELISA) plate reader (Biolab-System,Los Angeles, CA, USA). The percentage of replication was calculated from the optical density (OD) of each well as follows: ((mean OD of treated, infected neurons in well with drug)/(mean OD of non-treated and infected neurons)) × 100. Results are presented as the mean viability ± standard error of the mean (SEM).

### 2.9. Cytotoxicity Assay

To evaluate the cytotoxicity of neurons, we used the MTT assay. Progesterone and 17B-estradiol, along with their antagonists or agonists, were added to the neurons in vitro. The neurons were treated with E2 or P4 at 10, 20, 40, 80, and 160 nM/mL doses. In another assay, we added to the neurons E2 plus P4 at 160 nM/mL or E2 at 10, 20, 40, 80, and 160 nM/mL plus one µM/mL tamoxifen. We add progesterone at doses of 10, 20, 40, 80, and 160 nM/mL plus mifepristone at a 1 µM/mL concentration. PPT or DPN was added to the cells at a 1 nM/mL dose for 48 h.

### 2.10. Statistical Analysis

Quantitative variables are represented as the mean ± SEM. All the experimental groups were analyzed by analysis of variance (ANOVA) and a Dunnett T3 post hoc test. The control group consisted of uninfected cells that were 100% viable.

## 3. Results

### Step and Displacement Analysis

**Figure 1:**
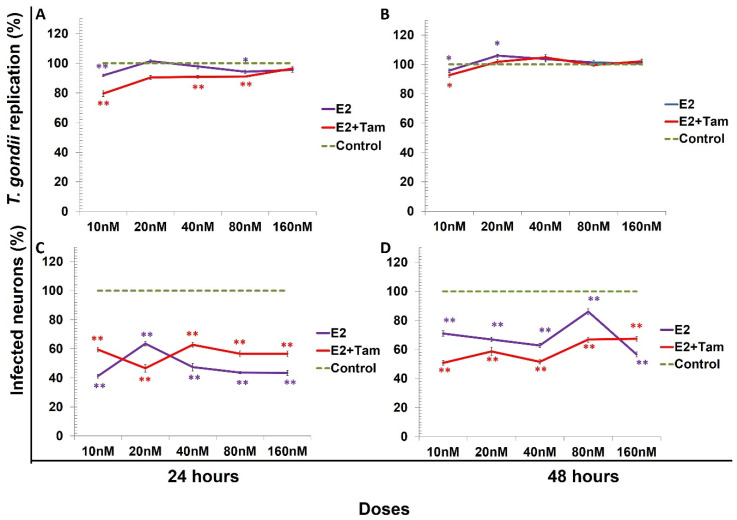
*Toxoplasma gondii* replication in neurons, representing the percentage of the control value. Untreated neurons (control; dashed line), neurons pretreated with 17β-estradiol (E2) (purple line), and neurons treated with E2 + tamoxifen (red line). 17β-Estradiol reduced *Toxoplasma gondii* replication at doses of 10 and 80 nM at 24 h. However, at 48 h, the replication was reduced only at the 10 nM dose and increased at 20 nM with a statistically significant difference of * *p* < 0.05. (**A**,**B**) The percentage of infected neurons was reduced by E2 and by the combination of tamoxifen at 24 and 48 h with statistically significant differences in respect to the control, ** *p* < 0.001; (**C**,**D**) exhibit these changes.

All data were compared vs. the control (100%). At 24 h, 17β-estradiol reduced approximately 10% *T. gondii* replication at doses of 10 and 80 nM, with a statistically significant difference (*p* < 0.001 and *p* < 0.005, respectively). The combination of E2 (10 nM) plus Tam reduced *T. gondii* replication by 20% with a statistically significant difference of (*p* < 0.001). We observed a reduction in *T. gondii* replication of 10% only with E2 at 10 nM and with E2 plus tamoxifen at a similar percentage with a significant difference compared to the control (*p* < 0.05) at 48 h (Figure 1A,B). The percentage of infected neurons treated with E2 was markedly reduced (58%) at 10 nM at 24 h. The combination of E2 at 10 nM plus Tam at one µM/mL decreased 50% the percentage of infected neurons at 48 h with a statistically significant difference of *p* < 0.001 (Figure 1C,D).

**Figure 2:**
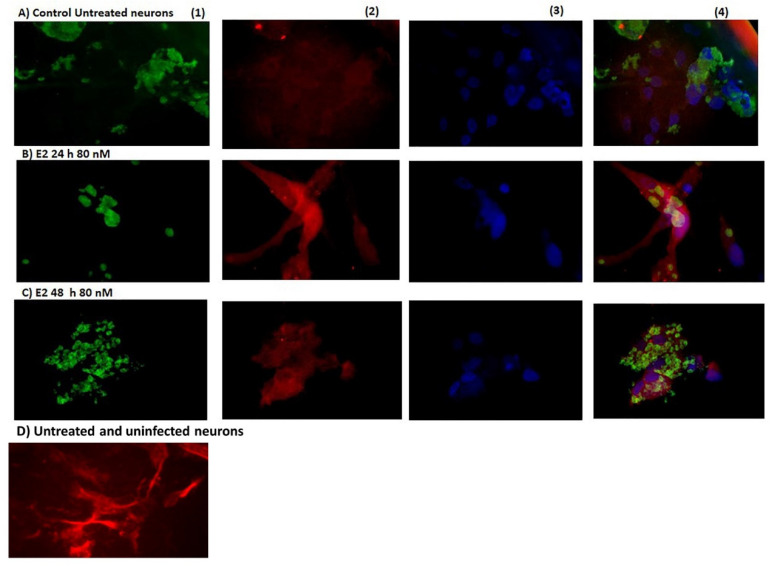
Representative photographs of *Toxoplasma gondii* infection in cultured neurons detected by immunocytochemistry. The parasite is shown in green; the neurons are presented in red. The neuron’s nucleus is in blue. The merged are depicted in the last column’s photographs. (**A**) The control group at 24 h showed the untreated and infected neurons (*T. gondii* tachyzoites RH strain). The neurons were destroyed almost completely by *Toxoplasma gondii*. (**B**) Observe that there was substantial damage to the neurons in the 17-β estradiol (E2)-treated group; however, some neuronal branches were conserved. (**C**) In the neurons treated with 17-β estradiol, the parasite changed its structure. (**D**) Negative control neurons, uninfected and untreated. The neuron exhibited some dendrites and did not show signs of damage. (40× magnification).

Figure 2 shows the morphology of infected neurons with *Toxoplasma gondii*. In (A) the positive control (untreated and infected neurons), *Toxoplasma* was in an aggregate form, and the neurons present were damaged. In (B), the structure of neurons infected by *Toxoplasma* and treated with E2 (80 nM) was more preserved than the control at 24 h. In (C), the neurons treated with E2 (80 nM) showed the highest infection by *Toxoplasma gondii* at 48 h.

**Figure 3:**
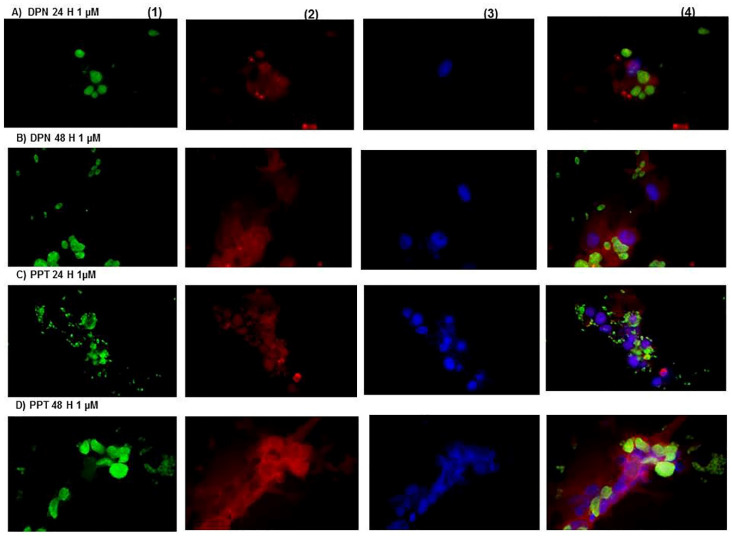
(**A**,**B**) (DPN 1µM, 24 and 48 h): The parasite destroyed the neurons treated with the agonist of ER β or DPN, in consequence, a significant number of the parasites were present at 24 and 48 h. (**C**,**D**) (PPT 1 µM, 24 and 48 h, respectively): The neurons treated with PPT had a more significant infection than those with DPN; however, the neurons were better preserved. (40× magnification).

In Figure 3A,B, the neurons infected with *Toxoplasma gondii* under the effect of DPN (1 nM/mL) at 24 and 48 h exhibited parasites intra- and extracellularly. In (C) and (D), the neurons in the presence of PPT (1 nM/mL) at 24 h and 48 h were more infected concerning DPN. The neurons were damaged, and a remarkable percent of them were destroyed.

**Figure 4:**
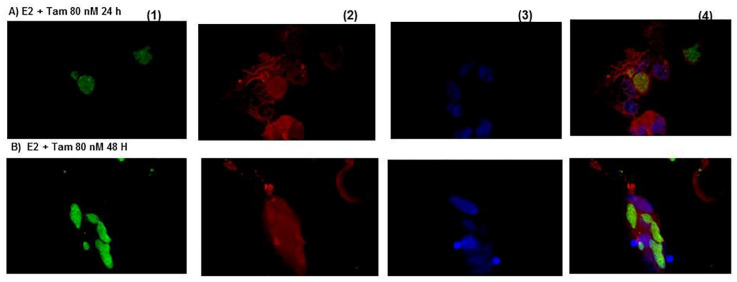
Representative photographs of *Toxoplasma gondii* infection in cultured neurons detected by immunocytochemistry. The parasite is in green, the neurons are presented in red, and the neuron’s nuclei are in blue. The merged are shown in the last column’s photographs. (**A**,**B**). Neurons treated with 17-β estradiol plus tamoxifen were destroyed and were less infected at 24 h than at 48 h compared to the untreated control. (40× magnification).

Figure 4A,B show neurons in the presence of E2 plus tamoxifen exhibiting a minor intracellular infection compared to E2 or PPT at 48 h. However, the neurons disintegrated and their cytoplasmic membrane was broken at 24 h of treatment.

**Figure 5:**
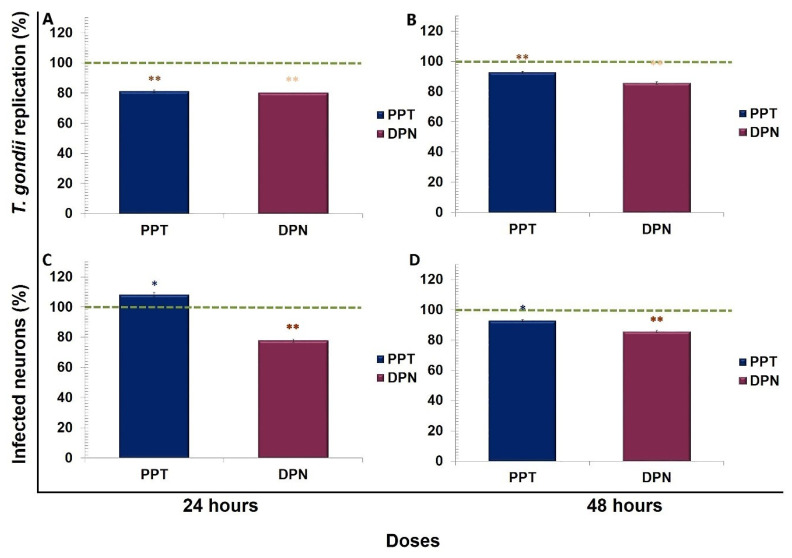
Percentage of *Toxoplasma gondii* replication at 24 h and 48 h. Percentage of *T. gondii*-infected neurons at 24 and 48 h after treatment with PPT (navy blue bar) or DPN (burgundy bar). (**A**,**B**) *T. gondii* replication was reduced by PPT and DPN with respect to the control (100%), with statistically significant differences of (*p* < 0.001). (**C**) At 24 h, the percentage of infected neurons treated with PPT was significantly increased, compared with the control (*p* < 0.05); however, DPN reduced this percentage significantly (*p* < 0.001). (**D**) At 48 h, the percentage of infected neurons was reduced by both agonists (i.e., PPT and DPN) with statistical significance compared with the control at * *p* < 0.05 and at ** *p* < 0.001, respectively.

PPT and DPN decreased *T. gondii* replication by approximately 20% compared to the control with a statistically significant difference of (*p* < 0.001) at 24 and 48 h p.i. (Figure 5A,B). The agonist of ERβ DPN reduced the percentage of infected neurons at 24 and 48 h (22% and 16%, respectively) versus the control with a statistically significant difference of (*p* < 0.001) (Figure 5C,D).

**Figure 6:**
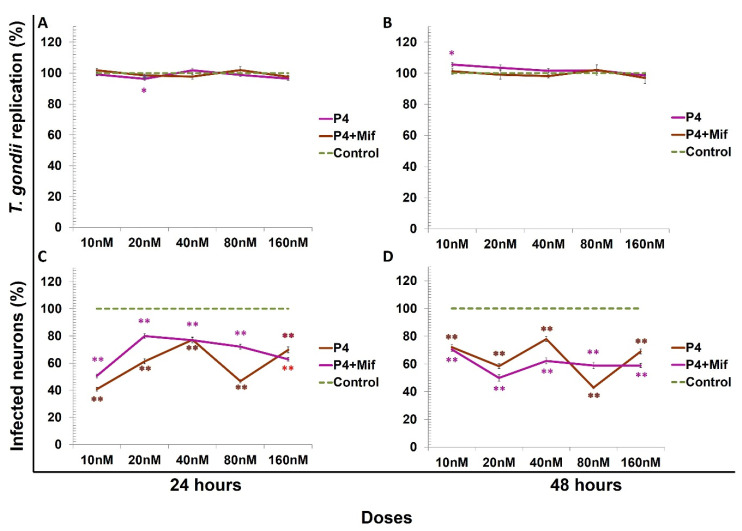
*Toxoplasma gondii* replication in neurons. Replication is expressed as the percentage of the control value. Untreated neurons (control; dashed line), neurons pretreated with progesterone (P4) (brown line), and neurons treated with P4 + mifepristone (fuchsia line). Progesterone did not regulate *Toxoplasma* infection, except at 20 nM dose by 24 h. However, at 48 h at a 10 nM dose, this replication increased with statistical significance: * *p* < 0.05 (**A**,**B**). (**C**,**D**) The percentage of infected neurons was reduced by progesterone combined with Mif at 24 and 48 h with a statistically significant difference of ** *p* < 0.001.

*Toxoplasma gondii* replication decreased by progesterone, only at a dose of 20 nM at 24 h. The combination of progesterone plus Mif (1 µM/mL) did not affect *T. gondii* replication (Figure 6A,B). There was a significant reduction in the percent of infected neurons by progesterone or by its combination with Mif (*p* < 0.001) both at 24 and at 48 h p.i. (Figure 6 C,D).

**Figure 7:**
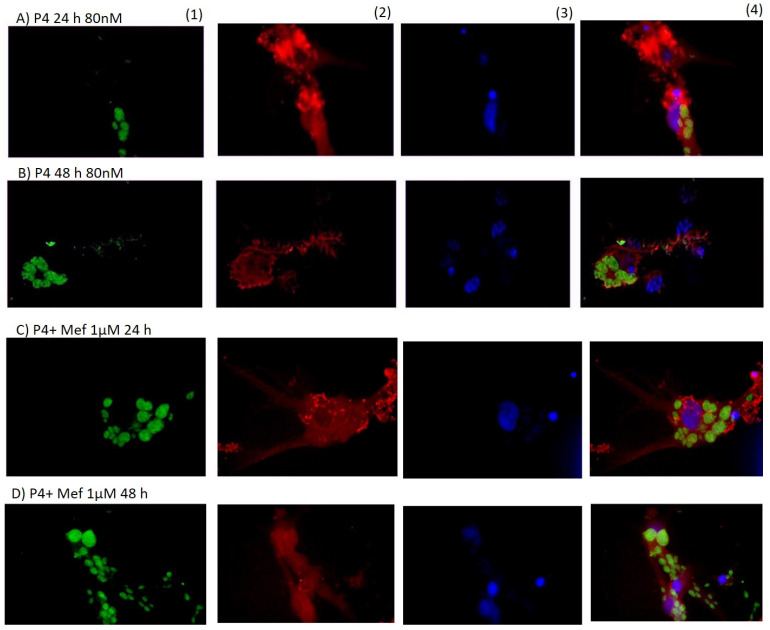
(**A**,**B**) Neurons infected with tachyzoites of *T*. *gondii* and treated for 24 and 48 h with progesterone at 80 nM. In (**C**,**D**) the neurons treated with progesterone plus mifepristone at 24 and 48 h exhibit many tachyzoites; however, the neurons present were better preserved compared to progesterone alone. (40× magnification).

Figure 7A,B, show that the neurons treated with progesterone at 24 and 48 h had more damage by *Toxoplasma* than E2 plus Tam or PPT or DPN. In Figure 7C,D, neurons infected and treated with progesterone and Mifepristone were better preserved than those treated only with progesterone.

**Figure 8:**
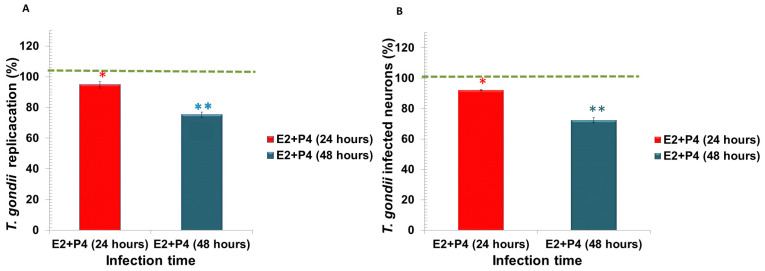
Percentage of *Toxoplasma gondii* replication treated with 17β-estradiol combined with progesterone E2 + P4 (red and blue bars, respectively) at 24 and 48 h. (**A**) Percentage of *T. gondii* in neurons decreased with E2 + P4, compared with the control, at 48 h p.i., ** *p* < 0.001. (**B**) Percentage of infected neurons treated with E2 + P4 was reduced compared with the untreated control at 24 and 48 h, with a significant difference of * *p* < 0.05 and ** *p* < 0.001, respectively.

The effects of E2 plus P4 on *Toxoplasma gondii* replication and the percentage of infected neurons are shown in Figure 8A,B. The pretreatments reduced the percentage of replication and neurons infected with E2 plus P4 (25–30%) at 24 and 48 h with a statistically significant difference of * *p* < 0.05 and ** *p* < 0.001, respectively.

## 4. Discussion

To our knowledge, this is a pioneer study in the analysis of the effect of hormonal steroids (i.e., 17β-estradiol and progesterone) as well as its agonists and antagonists regarding *Toxoplasma gondii* infection in cultured cortex neurons.

In brain mice, *Toxoplasma gondii* tachyzoites invade the microglia and the neurons [42,43]. In primary cultures of human fetal cells, *T. gondii* tachyzoites were replicated in astrocytes and neurons; the highest *T. gondii* infection was observed in astrocytes [44,45]. In the present study, neurons treated with E2 at a physiological dose (10 nM) decreased *T. gondii* replication at 24 h p.i. The highest concentration of E2 (160 nM) did not control the *Toxoplasma* infection. A saturation process could be taking place in the estradiol receptors. However, the percentage of infected neurons decreased at all doses of estradiol utilized in this study. 17β-Estradiol regulates the invasion of *Toxoplasma gondii* into neurons and may avoid the entry of parasites. However, a greater number of studies are necessary to corroborate this hypothesis. Tamoxifen in combination with estradiol reduced *T. gondii* replication in neurons even with a lower E2 dose; maybe the mechanism involves preferment binding of tamoxifen to ERβ compared to ERα, similar to what occurs with the DPN agonist. Tamoxifen, as a SERM member, competes with estradiol: however, its affinity for classical estrogen receptors is minor to E2, and it could act as a partial agonist [26,46]. Pung and Luster 1986 [36] demonstrated that tamoxifen could reduce *Toxoplasma gondii* cysts in mouse brain. In this study, we observed a reduction in *Toxoplasma* tachyzoites inside neurons by tamoxifen; this effect was similar in astrocytes infected by *Toxoplasma gondii* [47]. On the other hand, it has been reported that tamoxifen induces autophagy in infected cells, and this may be another mechanism involved in reducing *Toxoplasma gondii* burden in neurons [48,49].

The interaction of E2 and Tam also decreased the percentage of infected neurons. Our results are similar to those found by Zhang in 2017, who showed that tamoxifen reduces the number of parasites by approximately 50% in host cells [50].

**Figure 9:**
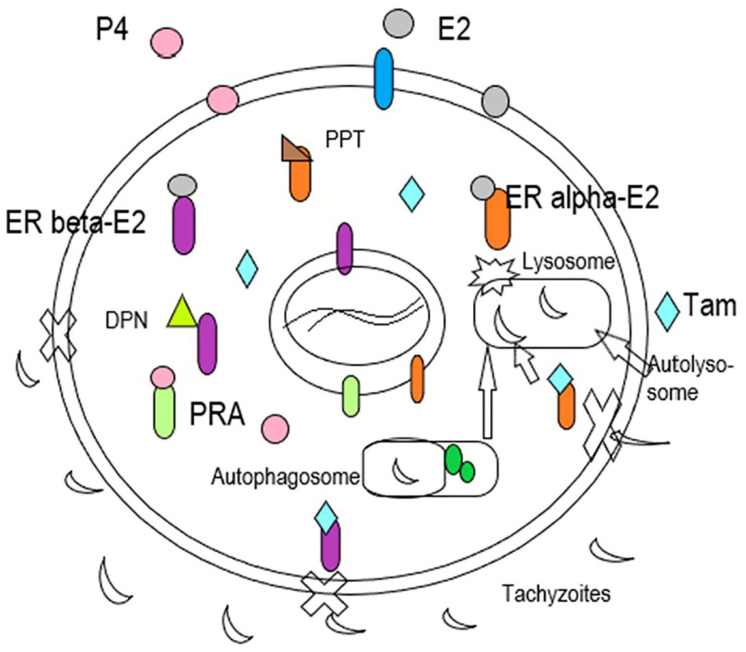
The interactions among sex hormones (i.e., estradiol, progesterone) and tamoxifen (Tam) with the tachyzoites of *Toxoplasma gondii*.

The mechanism by which tamoxifen affects *Toxoplasma* infection is unknown. However, in Figure 9, we discuss the possible interactions of sex hormones with *T. gondii* infections.

Observe that tamoxifen can induce autophagy and the steroid hormones can avoid the entrance of parasites to neurons through the membrane. The mechanism of this last process is unknown. Figure 9 shows the interactions between hormones, and their receptors are shown on its specific receptor. Tamoxifen binds to ERβ (blue and purple), and it is possible that this complex, induces the formation of an autophagosome. With the presence of proteins (in green) necessary for this process, in consequence, it will active a lysosome and will form an autolysosome. Progesterone (P4, pink), receptor of progesterone (PRA, light green), E2 (estradiol, gray), estradiol receptor beta (ER-beta-E2, gray and purple), selective agonist for receptor β of E2, 23-bis(4-hydroxyphenyl) propionitrile (DPN, green triangle). Estrogen receptor α bind to estradiol (ER-alfa-E2, orange and gray), selective agonist of receptor α, E2 4,4′,4″-(4-propyl-[1H] pyrozole-1,3,5-triyl) trisphenol (PPT, brown triangle ), selective estrogen receptor modulator, tamoxifen (Tam, blue rhombus).

In the present study, DPN produced an essential reduction in *Toxoplasma gondii* infection in neurons in vitro. The response of the neurons to this agonist maybe is due to fact of its high specificity for ERβ, affording better neuronal protection [51,52,53,54,55].

Progesterone did not reduce *Toxoplasma gondii* replication despite; it decreased the percentage of infected neurons. Progesterone could modulate the number of their receptors in neurons in vitro [55]. In current studies of our group of work, we reported that progesterone could reduce ERβ and increase ERα in THP1 cells, maybe in neurons could be a similar regulation and thus modulate the parasite invasion [39].

The antagonist of progesterone, Mif, did not revert the action of progesterone on *T. gondii* replication at any of the experimental dosages; this could be due to the dose and time of exposure. However, there are no previous studies in this regard; thus, it is imperative to acquire a more significant number of investigations on this topic. The combination of progesterone and Mif exerted a lesser effect on *Toxoplasma* infected neurons compared with progesterone alone. One explanation could be a change in progesterone membrane receptors as well as in their affinity [13,30,31,32].

The combination of 17β-estradiol and progesterone significantly reduced *Toxoplasma gondii* replication and the percentage of infected neurons; this may be due to the major expression of classical estrogen receptors [39], E2 plus progesterone modulating the viability of extracellular parasites and the passive invasion to neurons. This result is similar to host cells infected with tachyzoites treated with dehydroepiandrosterone (DHEA) [13,56,57].

## 5. Conclusions

E2 and tamoxifen as well as PPT and DPN regulated *Toxoplasma gondii* infection in neurons.

The neuronal response to these agonists is clear evidence of the essential participation of classical estrogen receptors in the process of *Toxoplasma gondii* infection.

The combination of E2 and P4 decreased *Toxoplasma gondii* infected neurons. This action could be considered as a neuro-protector effect on *T. gondii* infection.

## Data Availability

Not applicable.
